# Corrigendum to “Role of remote sensing, geographic bioinformatics system and bioinformatics in kala-azar epidemiology” [originally published as J Biomed Res 2011, 25(6):373-384; doi:10.1016/S1674-8301(11)60050-X]

**Published:** 2012-09

**Authors:** Gouri Sankar Bhunia, Manas Ranjan Dikhit, Shreekant Kesari, Ganesh Chandra Sahoo, Pradeep Das

**Affiliations:** Rajendra Memorial Research Institute of Medical Sciences (ICMR), Agamkuan, Patna, Bihar 800007, India.

The author would like to inform the readers that the correct form of title “Role of remote sensing, geographic bioinformatics system and bioinformatics in kala-azar epidemiology” , content in abstract “The computational approaches like remote sensing, geographic information system (GIS) and bioinformatics are the key re-sources for the detection and distribution of vectors, patterns, ecological and environmental factors and genomic and proteomic analysis.”, and keyword “geographical bioinformatics systems (GIS)” should be read as follows: title “Role of remote sensing, geographical bioinformation system and bioinformatics in kala-azar epidemiology”, content in abstract “The computational approaches like remote sensing, geographical information system (GIS) and bioinformatics are the key re-sources for the detection and distribution of vectors, patterns, ecological and environmental factors and genomic and proteomic analysis.”, and keyword “geographical bioinformation system (GIS) ”.

We apologize for any inconvenience this error may have caused.

